# Purifying selection leads to low protein diversity of the mitochondrial *cyt* b gene in avian malaria parasites

**DOI:** 10.1186/s12862-023-02155-5

**Published:** 2023-09-11

**Authors:** Xinyi Wang, Staffan Bensch, Xi Huang, Lu Dong

**Affiliations:** 1 MOE Key Laboratory for Biodiversity Science and Ecological Engineering, College of Life Sciences, Beijing Normal University, Beijing, 100875 China; 2 Department of Biology, Molecular Ecology and Evolution Laboratory, Lund University, Ecology Building, 223 62 Lund, SE Sweden

**Keywords:** Purifying selection, Cytochrome B, Host specificity, Avian malaria

## Abstract

**Background:**

Mitochondrial respiration plays a central role in the survival of many eukaryotes, including apicomplexan parasites. A 479-bp fragment from the mitochondrial cytochrome b gene is widely used as a barcode to identify genetic lineages of avian malaria parasites *Plasmodium* and related haemosporidians. Here we looked for evidence of selection in the avian *Plasmodium cyt* b gene, using tests of selection and protein structure modeling. We also tested for the association between *cyt* b polymorphism and the host specificity of these parasites.

**Results:**

Based on 1,089 lineages retrieved from the Malavi database, we found that the frequency of the most conserved amino acids in most sites was more than 90%, indicating that the protein diversity of the avian *Plasmodium cyt* b barcode was low. The exceptions were four amino acid sites that were highly polymorphic, though the substitutions had only slight functional impacts on the encoded proteins. The selection analyses revealed that avian *Plasmodium cyt* b was under strong purifying selection, and no positively selected sites were detected. Besides, lineages with a wide host range tend to share *cyt* b protein haplotypes.

**Conclusions:**

Our research indicates that purifying selection is the dominant force in the evolution of the avian *Plasmodium cyt* b lineages and leads to its low diversity at the protein level. Host specificity may also play a role in shaping the low mitochondrial diversity in the evolution of avian malaria parasites. Our results highlight the importance of considering selection pressure on the *cyt* b barcode region and lay a foundation for further understanding the evolutionary pattern of mitochondrial genes in avian malaria.

**Supplementary Information:**

The online version contains supplementary material available at 10.1186/s12862-023-02155-5.

## Background

In most eukaryotes, mitochondria play a central role in cellular metabolism, providing the energy necessary for survival. Among them, apicomplexan parasites, which obtain nutrition through obligate intracellular parasitism, have a highly reduced mitochondrial genome. Their mitochondrial genome is the smallest among all known eukaryotes, composed of three protein-encoding genes: cytochrome b (*cyt* b), cytochrome oxidase I (*cox1*), and cytochrome oxidase III (*cox3*) plus fragmented ribosomal RNAs [1–4], which signifies the reduced function of the organelle [5, 6].

Cytochrome b is the only mitochondrial gene that seems to be unconditionally necessary for the metabolism and survival of *Plasmodium* [7]. The coding product Cyt b occurs as part of the cytochrome III assembly in the electron transport chain (ETC). Cyt b contains eight transmembrane domains together with two functional reaction sites: ubiquinol oxidation site (Q_o_) and ubiquinone reduction site (Q_i_) [8]. Owing to its central role in cellular energy production, variable selective pressures in different host species may contribute to its evolutionary patterns [9].

In free-living eukaryotes, despite several contrary evidence, *cyt* b has been largely assumed to be under neutral evolution [10, 11]. However, the selection from diverse hosts implies that such a pattern may not hold for apicomplexan parasites. Given the important functions of *cyt* b and its current use as a barcode in some apicomplexan parasite taxa, it is necessary to investigate the diversity of *cyt* b and its possible driving forces. Earlier studies suggested that positive selection acted on *cyt* b barcode region of Haemosporidian parasites as a result of the switch from avian hosts to mammals [12, 13]. Pacheco et al. [14] proposed that the haemosporidian mitochondrial DNA (mtDNA) was under purifying selection and the different strengths of negative selection among parasite clades may be due to the co-evolution with adaptation to vector insects. In *Eimeria* parasites, positive selection was detected in several codons of mtDNA-encoded proteins against a background of purifying selection. Host species and pathogenicity were considered to be important factors that might shape mitochondrial variation [15].

Avian malaria parasites (genus *Plasmodium*) are a class of mosquito-transmitted haemosporidian parasites that cause infectious diseases. They are globally distributed [16–18], infecting over 2,400 avian species [19], and pose a serious threat to bird health [20]. A 479-bp segment of c*yt* b sequences has been widely used as a barcode to define unique lineages of avian malaria parasites and related haemosporidians [21, 22], which is included in the MalAvi database (http://mbio-serv2.mbioekol.lu.se/Malavi/index.html) [23]. Avian *Plasmodium* parasites are the basal clade of malaria parasites and have higher species diversity than other groups of malaria parasites [14], which makes it an ideal system for studying the molecular evolution of pathogens. However, the selection pressure of avian malaria *cyt* b evolution remains unknown and it is an open question to explore why the identical *cyt* b barcodes sometimes are shared by different cryptic species [24, 25]. In order to evaluate the availability of the *cyt* b barcode region, we investigated its selection pressure in avian malaria parasites.

The ability of avian malaria lineages to adapt to different hosts varies widely, reflected by the highly variable host range [18, 26]: More than half of avian malaria parasites appear to infect single host species, whereas other parasites could infect several or even dozens of bird species [27]. For instance, the notorious *Plasmodium relictum* has infected at least 120 species of birds around the world, leading to the extinction of a variety of Hawaiian land birds [28, 29]. Whether host specificity would act as a selection force on *cyt* b variation in avian *Plasmodium* remains to be studied. Previous studies generally focused on nucleotide diversity of *cyt* b [21, 27], but few on protein diversity, which limits our understanding of the driving forces of the evolution of avian malaria parasites.

In this study, we analyzed a total of 1,089 avian *Plasmodium* lineages recorded in the MalAvi database using an integrative approach combining tests of selection, amino acid polymorphism analysis, and protein 3D structure modeling. Specifically, we aim to: (i) present the protein haplotype diversity of avian *Plasmodium cyt* b, (ii) test for selection acted on the *cyt* b proteins, and (iii) explore the possible impact of host specificity on *cyt* b protein diversity in avian malaria parasites.

## Result

### Protein diversity of avian *Plasmodium* barcodes

**Fig. 1:**
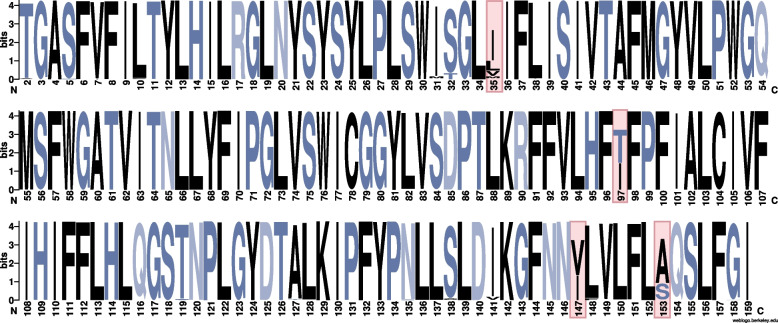
The relative frequencies of amino acids at each amino acid site of 474-bp *cyt* b. The plot is based on sequences of 1,089 avian malaria lineages. Sites whose most conserved amino acid accounted for no more than 75% are defined as highly polymorphic sites (HPSs) and are highlighted in pink. (The first amino acid was trimmed, see Methods)

A total of 459 protein haplotypes were acquired from 1,089 avian malaria lineages. When looking at each site across the 158 aa fragment separately, the frequency of the most conserved amino acid at 154 sites was above 90%, including seven fixed sites (Fig. 1). The remaining four sites (sites 35, 97,147, and 153) which have no amino acids with frequencies higher than 75%, was defined as highly polymorphic sites (HPSs) (Fig. 1, Additional file 1: Fig. S1). Even if in HPSs, the two most conserved amino acids together account for over 90% of the total samples.

### Protein modeling and mutation effects of highly polymorphic sites

**Fig. 2:**
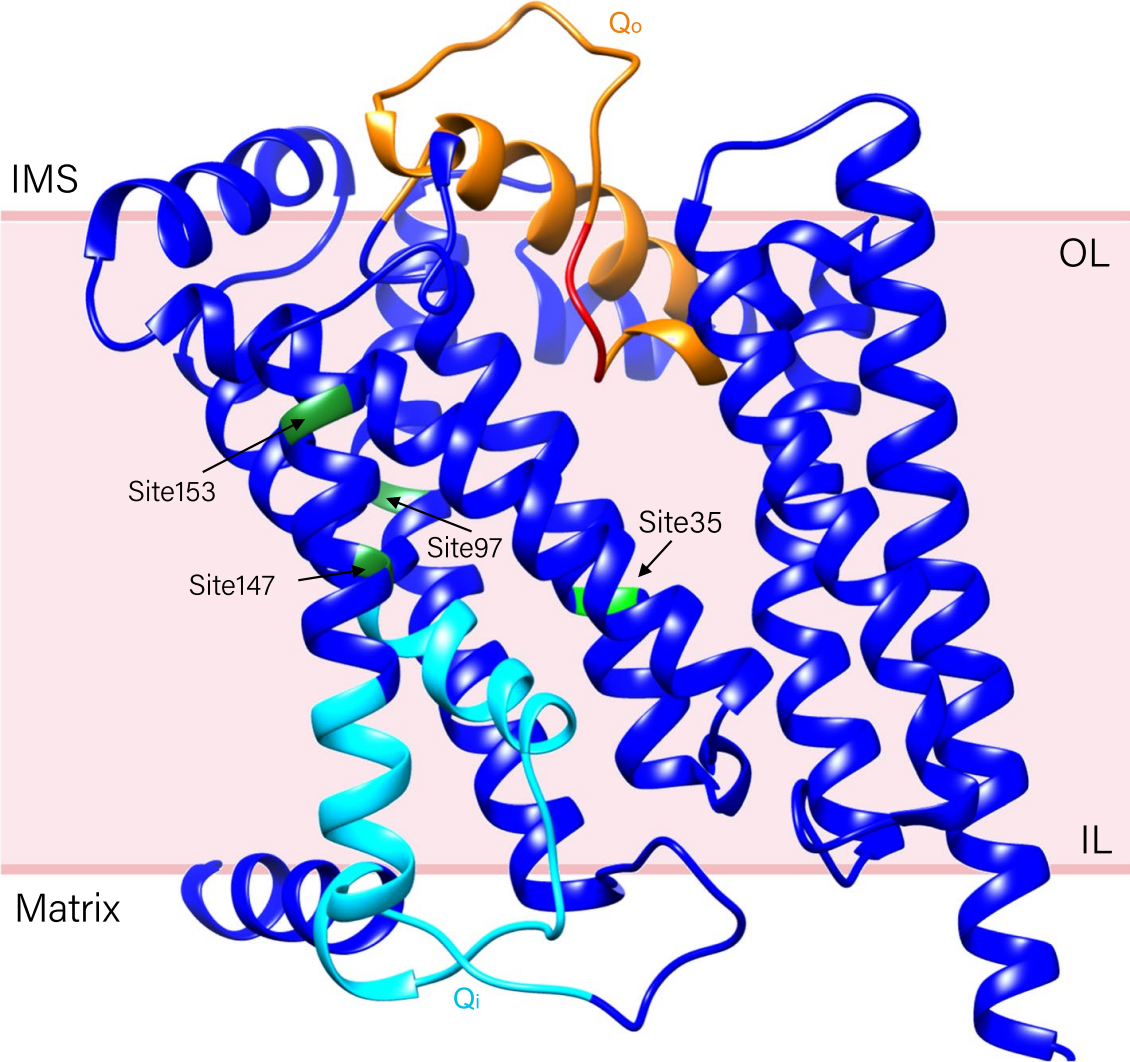
Three-dimensional ribbon model of CytB protein in avian malaria predicted by the three-track neural network. The mitochondrial inner membrane (MIM), as previously suggested [30], is roughly marked with two horizontal lines. The site numbers with arrows correspond to the positions in the barcode region as shown in Fig. 1. (Green: highly polymorphic sites, light blue: Qi residues, orange: Qo residues, red: the catalytic PEWY motif. IMS, inner membrane space; IL, inner leaflet; IMS, inner membrane space; OL, outer leaflet)

A three-dimensional protein model of full-length *cyt* b of *Plasmodium relictum* (Fig. 2) was generated with the confidence of 0.78 (1.0 good, 0.0 bad). HPSs were mapped onto the predicted 3D structure and all of them were located in the transmembrane domain. The PROVEAN analysis suggested that the substitutions occurring in all four HPSs were neutral (Table 1). Three sites (sites 35, 97, and 147) were identified as destabilizing indicating a gain in flexibility while site 153 showed a decrease in molecule flexibility by DYNAMUT (Table 1).

**Table 1:** The potential functional effect of main amino acid substitutions at HPSs (x—indicate positive selection by the selection test)

Site	Ancestral amino acid	Alternative amino acid	Domain	PAML	DATAMONKEY				Provean		DYNAMUT	
					SLAC	FEL	MEME	FUBAR	Score	Prediction (cutoff = -2.5)	ΔΔG(kcal/mol)	Action
35	Ile	Leu	Trans	-	-	-	x	-	-0.913	Neutral	0.372	Stabilizing
	Ile	Val	Trans						-0.370	Neutral	-0.371	Destabilizing
97	Thr	Ile	Trans	-	-	-	-	-	2.683	Neutral	1.045	Stabilizing
147	Val	Ile	Trans	-	-	-	-	-	0.396	Neutral	0.677	Stabilizing
153	Ala	Ser	Trans	-	-	-	-	-	-0.776	Neutral	-1.088	Destabilizing

### Evidence of natural selection

The *cyt* b barcode region presented a high percentage of codons under purifying selection, according to the SLAC, FUBAR, and FEL methods. The majority of codons were under purifying selection (122,128, and 128 sites, respectively), together with 20 neutral codons. No evidence of positive selection was observed by SLAC, FUBAR, and FEL tests (Table 2). Four codon positions (sites 35, 44,136, and 143) were suggested to be under positive selection by MEME (Table 2). In the site model analyses of CodeML, the null model was rejected in the pairwise comparisons indicating that the ω values were variable across sites and *cyt* b was not evolving under neutrality (Additional file 1: Table S1). Though positive selection models (M2a / M8) fitted the data better than the nearly neutral models (M1a / M7), no positive selection sites were detected.

**Table 2:** Results of selection tests according to five different methods

Methods	Positive selection sites
SLAC	/
FEL	/
FUBAR	/
MEME	sites 35, 44, 136, 143
PAML	/

“/” indicates no positively selected sites were detected

### The correspondence between DNA sequences and their protein haplotypes

We found that 61 protein haplotypes were shared by 65.3% of the lineages (*n* = 711) while 378 lineages possessed unique protein haplotypes. Among 980 lineages with bird host information, 651 lineages shared 61 protein haplotypes, and 319 lineages had unique protein haplotypes (Additional file 1: Fig. S2). The point-biserial correlation coefficient was positive (*r* = 0.173, *P* < 0.001), suggesting that parasites that can infect more host species tend to share the *cyt* b protein haplotypes with others.

## Discussion

In the current study, we demonstrated that purifying selection is the main evolutionary force leading to low *cyt* b protein diversity. Additionally, host specificity may be a factor in shaping the mitochondrial diversity in avian malaria parasites. These results enhance our understanding of the molecular evolution of avian malaria *cyt* b.

### Diversity and selection of the *cyt* b barcode region in avian malaria parasites

The frequency of the most conserved amino acids in almost all sites was more than 95%. Moreover, all four HPSs were located in the transmembrane domain rather than the functional reaction sites Q_o_ or Q_i_. Such non-synonymous substitutions seemed to have only slight, if any functional effects. These patterns indicate that the protein diversity in *cyt* b barcode region of avian malaria parasites was low.

According to four different DATAMONKEY selection tests, a high number of purifying selection sites were observed with the exception of a few positively selected sites detected by MEME. In PAML, M2a (selection) / M8 (beta&ω) fitted the data better than M1a (neutral) / M7 (beta), indicating that this gene was not evolving under neutrality. Additionally, no positive selection sites were detected under M2a and M8. Sites that had received consistent support in at least two methods can be considered as positive selection sites (as recommended by [31, 32]). Hence, we hold that avian *Plasmodium cyt* b was globally evolving under purifying selection and there were no sites under positive selection*.* Indeed, given the key function in pyrimidine biosynthesis in the asexual stage, and an ATP-generating function in the insect stage [6, 33], it’s not surprising that purifying selection is the predominant force shaping *cyt* b evolution, which is in agreement with the general trends in mtDNA selection tests [14, 31]. Moreover, computer simulations suggested that the efficiency of purifying selection in *Plasmodium* was intensified by their complicated life cycle [34], which was consistent with our observations from actual data. Purifying selection constantly sweeps away deleterious mutations that are produced in each generation to ensure functional stability and thus plays a key role in shaping the low protein diversity of avian *Plasmodium cyt* b*.*

### The possible impact of host specificity on *cyt* b protein diversity

In the analyses of the correspondence between lineages and their protein, we found that about two-thirds of lineages shared *cyt* b haplotypes with others, while the rest of the lineages possessed unique protein haplotypes. An important question is whether the lineages sharing protein haplotypes have some phenotypic or life-history characteristics in common. There is limited phenotypic information related to the mtDNA [14]. However, host diversification seems to be one of the important factors that may shape mitochondrial variation in parasites. Haemosporidian *cyt* b lineages and their avian hosts show evidence of a weak but significant cophylogenetic congruence [35], while they are still capable of switching to phylogenetically distant hosts. In *Eimeria*, another class of apicomplexan parasites, their mtDNA changes may be adaptive, driven by complex interactions with their host species [15]. Interestingly, we found that parasites with a broad host range (generalist) tend to share *cyt* b protein haplotypes with others. In some cases, phylogenetically related generalist lineages shared protein haplotypes (Additional file 1: Fig. S3), which may be largely shaped by the neutral process in the early stage of speciation. Generalist *Plasmodium* parasites are typically apical in phylogenies [36], thus there is not enough time to accumulate non-synonymous mutations on small evolutionary timescales, leading to little or no differentiation in protein sequences. Besides, we also observed the pattern that phylogenetically distant generalist lineages share protein haplotypes (Additional file 1: Fig. S3). It may be due to the convergent evolution at molecular level and may imply the potential for homoplasy. Although here we did not detect directly relevant adaptive sites in the partial *cyt* b, we surmise that if there are adaptive sites elsewhere but linked to this fragment, a similar pattern would also appear. It should be noted that sequencing errors of the *cyt* b barcodes may bias the signal we found here. An overlooked sequencing error will in most cases be assigned as a new lineage and by necessity end up as an artifact. In addition, although the sampling effort has expanded much to include more regions and different host species in recent years, the geographic and host taxonomy sampling bias remains in our dataset and may lead to some underestimation of the host range.

### Availability of *cyt* b as the barcode of avian malaria parasites

Defining taxonomic units is the prerequisite to understanding the distribution, speciation, and transmission [19] of avian malaria parasites. This issue has been under continuous discussion, yet we remain ambiguous about it [37]. At present, owing to the multiple challenges (especially host contamination) in obtaining haemosporidian genomic data [38], *cyt* b is the most widely used barcode to define avian malaria lineages. A haplotype with one or more nucleotide differences from existing lineages will be defined as new.

Is *cyt* b still suitable as the barcode of avian malaria parasites after observing the above molecular evolution pattern? On the one hand, we should be cautious that the mutation rate of *cyt* b in *Plasmodium* is much slower than in animals in general [39]. Purifying selection sweeps away deleterious mutations and thus reduces the diversity of avian *Plasmodium cyt* b*.* In addition, generalist parasites may be under certain evolutionary constraints and seem to have more similarities in their *cyt* b barcode region than specialist parasites. On the other hand, *cyt* b has many advantages as a barcode. We did not detect any positively selected sites in the *cyt* b barcode region. Though *cyt* b was under strong purifying selection at the protein level, the nucleotide haplotypes show much higher diversity than species based on morphological characters and/or host taxa [18]. For more than half a century, it is generally believed that synonymous mutations are neutral or near neutral, but see [40]. Besides, *cyt* b gene trees and parasite species trees bear a reasonable correspondence [37]. Taken together, we hold the view that cyt b is still the most useful marker to define lineages at present.

Given the low genetic diversity in *cyt* b gene, it would be better to delimitate phylogenetic species based on more fast-evolving loci. Thus, we need to employ the whole mitochondrial genome or more novel nuclear markers to better estimate the level of genetic diversity and species phylogenies [27] of avian malaria parasites.

## Conclusions

In summary, our work showed that purifying selection is the dominant force in the evolution of the avian *Plasmodium cyt* b and leads to its low protein diversity. Although the *cyt* b gene has been considered to be a good marker for finding and typing malaria parasites in general, the impact of using *cyt* b as a barcode on our understanding of avian malaria diversity remains to be discussed. Our study also provided a new perspective for understanding the vital question ‘What makes a generalist a generalist?’ in malaria research based on the function and evolution of mitochondrial protein. Nevertheless, barcoding sequences are not powerful enough if we want to further understand the evolutionary mechanism of host specificity of haemosporidian parasites. At present, genomic data of avian haemosporidian have proved highly difficult to generate [38] and for the vast majority of lineages, no nuclear genes have been studied. Improvements in sequencing and assembly technology are needed to proceed future genomic data accumulation of a wide range of haemosporidian in an attempt to bring insights into the evolution of avian malaria parasites.

## Methods

### Data collection

We retrieved all the avian *Plasmodium* 479-bp lineage sequences from the MalAvi database (v2.5.5, accessed on Nov 7, 2022) [23] and performed data filtering in two steps. First, the sequences of lineages were trimmed to 474-bp by removing the first three and last two nucleotides, because many lineages miss the first nucleotide and the last two nucleotides do not cover a full codon. To ensure the integrity of protein sequences, only lineages with the whole 474-bp sequences were retained. Lineages containing ambiguous bases were considered as unresolved mixed infections and removed from our dataset. Second, nucleotide sequences were translated into amino acid sequences with protozoan mitochondrial genetic code using MEGA v11 [41]. Lineages sharing the same *cyt* b protein sequence were regarded as having the same *cyt* b protein haplotype. In this step, five lineages (PIPFAS04, ICTCAY03, ICTCAY04, PASVER01, and PASVER02) with an unexpected stop codon were deleted. Lineages that passed the above screening constitute the final dataset (Additional file 2). The number of avian host species was calculated from the ‘Hosts and Sites Table’ in the MalAvi database.

### Sequence polymorphism and protein structure

We visualized the *cyt* b protein diversity pattern and represented the proportion of different amino acids at each site in all samples using WebLogo (http://weblogo.berkeley.edu) [42]. Sites whose most conserved amino acid accounted for no more than 75% were defined as highly polymorphic sites (HPSs). In order to test whether the amino acid changes in HPSs have an effect on the *cyt* b protein, we build a three-dimensional protein model of full-length *cyt* b of *Plasmodium relictum* and map HPSs onto it in four steps. First, we downloaded the full-length amino acid sequence of *Plasmodium relictum* (lineage: GRW11) *cyt* b [GenBank: ATD12902.1] [14] as the reference for mapping and predicted the protein structure using RobeTTaFold (https://robetta.bakerlab.org). This approach uses a three-track neural network and has shown to be one of the best performing public servers for predicting protein structure [43]. Second, the predicted protein structure was then annotated and recolored using Chimera (UCSF, www.rbvi.ucsf.edu/chimera) [44]. Third, we identified Q_o_ and Q_i_ sites as previously described by [45]. Finally, we marked all the HPSs onto the protein model.

The potential functional effect of amino acid substitutions was assessed by the Protein Variation Effect Analyser (PROVEAN: http://provean.jcvi.org/index.php, [46]. We used the default cutoff “-2.5” for high balanced accuracy. Furthermore, the impact of mutations on protein stability and dynamics was evaluated by the DYNAMUT web server (http://biosig.unimelb.edu.au/dynamut/, [47]). The reconstruction of ancestral sequences was conducted by the FASTML web server (http://fastml.tau.ac.il/) based on the phylogenetic relations between homologous sequences [48].

### Selection analysis

Selection at specific amino acid positions was assessed by multiple methods. Sites that had received consistent support in at least two methods can be considered as positively selected sites (PSSs) [31, 32]. As the length of the barcode region is relatively short, the big sequence set would reduce the reliability of the phylogenetic tree, and then affect the accuracy of the selection analysis. Here we extracted 460 lineages of 479-bp as a subsample for selection analysis. Four codon models implemented on the DATAMONKEY web server ( http://www.datamonkey.org/) [49] were used to assess codons under positive or purifying selection, including Single Likelihood Ancestral Counting (SLAC) [50], Fast Unconstrained Bayesian AppRoximation (FUBAR) [51], Fixed Effects Likelihood (FEL) and Mixed Effects Model of Evolution (MEME) [52]. The GTR model was used as the best substitution model in the above tests. Furthermore, we calculated the ratio of non-synonymous (d_N_) to synonymous sites (d_S_) using the site model of CodeML, implemented in PAML 4.9 [53]. The following six models were compared in pairs by Likelihood-ratio tests (LRT): M0 (one ω ratio), M1a (nearly neutral), M2a (positive selection), M3 (discrete), M7 (beta), and M8 (beta & ω). The Bayes empirical Bayes (BEB) was used to identify sites under positive selection [54].

The Bayesian tree used in the selection tests was reconstructed using BEAST v2.6.0 [55], applying the model GTR + G, which was suggested as fit best for the dataset in jModelTest v2.1.10 [56] according to the Bayesian inference criterion (BIC). The Markov chain Monte Carlo (MCMC) was set to ten initialization attempts, with the length of the chain as 1 × 10^8^ and tree log parameters as every 1 × 10^4^ generations. Tracer v1.7.1 (http://tree.bio.ed.ac.uk/software/tracer/) was used to measure the convergence of MCMC chains. After deleting the first 1,000 trees as burn-in, the maximum credibility tree was estimated by TreeAnotator v1.7.5 and visualized in Figtree v1.4.4 (http://tree.bio.ed.ac.uk/software/figtree/). In addition, TCS haplotype networks [57] of lineages that had identified morphological species were processed by PopART v1.7 software [58].

### Statistical analysis

Basic data collation was implemented through Microsoft Excel and Python scripts. To explore the relationship between host range and the diversity of protein haplotypes of avian malaria lineages, we first visualized the correspondence between lineages and their protein haplotypes using Sankey diagrams by SankeyMATIC (https://sankeymatic.com/build/). Then, a point-biserial correlation was calculated using the built-in function cor. test() in R v. 4.0.3 (https://www.r-project.org), to test whether lineages with a wider host range tend to share *cyt* b protein haplotypes with other lineages. Lineages exclusively obtained from insects (*n* = 119) were excluded from this analysis as they lack information on avian hosts. The independent variable was continuous which represented the host range of an avian malaria lineage. The dependent variable was binary: lineages that share protein haplotypes with others were defined as ‘1’, while those having unique protein haplotypes were defined as ‘0’.

### Supplementary Information

**Additional file 1: Figure S1. **The frequency of the dominating amino acid at each amino acid site of 474-bp *cyt *b (the first amino acid was trimmed, see methods). The plot is based on sequences of all 1,089 avian *Plasmodium* lineages. HPSs are highlighted in pink. **Figure S2.** The correspondence between *cyt* b (right side) and their protein haplotypes (left side) of avian malaria parasites. The thickness of the line indicates the size of host range. **Figure S3.** A sub-clade of the avian malaria lineages that share the same protein haplotype (pCYTB1) by phylogenetically related lineages (ALARV04, GRW04, GRW11, PHCOL01, and SGS1) and a distant lineage (SW5). **Table S1.** Results of PAML analyses testing for selection on the 479-bp *cyt* b.

**Additional file 2.**

## Data Availability

Original datasets are freely available via Malavi (http://130.235.244.92/Malavi/). The accession number of lineages in the final dataset is available via Additional file 2.

## Abbreviations

BEB: Bayes Empirical Bayes
BIC: Bayesian inference criterion
*cox1*: Cytochrome oxidase 1
*cox3*: Cytochrome oxidase 3
*cyt* b: Cytochrome B
dN: Number of non-synonymous substitutions per non-synonymous sites
dS: Numbers of synonymous substitutions per synonymous sites
ETC: Electron transport chain
FEL: Fixed Effects Likelihood
FUBAR: Fast Unconstrained Bayesian AppRoximation
GTR: General Time Reversible
HPSs: Highly polymorphic sites
LRT: Likelihood ratio test
MEME: Mixed Effects Model of Evolution
MIM: Mitochondrial inner membrane
mtDNA: Mitochondrial DNA
PSSs: Positively selected sites
Q_o_: Ubiquinol oxidation site
Q_i_: Ubiquinone reduction site
SLAC: Single Likelihood Ancestral Counting
