# Supplementary material for: Purifying selection leads to low protein diversity of the mitochondrial cyt b gene in avian malaria parasites
Source: BMC Ecol Evol. 2023 Sep 11;23:49. doi: 10.1186/s12862-023-02155-5 (PMC10494422; doi:10.1186/s12862-023-02155-5)
Supplement: Supplementary file 1 — Additional file 1: Figure S1. The frequency of the dominating amino acid at each amino acid site of 474-bp cyt b (the first amino acid was trimmed, see methods). The plot is based on sequences of all 1,089 avian Plasmodium lineages. HPSs are highlighted in pink. Figure S2. The correspondence between cyt b (right side) and their protein haplotypes (left side) of avian malaria parasites. The thickness of the line indicates the size of host range. Figure S3. A sub-clade of the avian malaria lineages that share the same protein haplotype (pCYTB1) by phylogenetically related lineages (ALARV04, GRW04, GRW11, PHCOL01, and SGS1) and a distant lineage (SW5). Table S1. Results of PAML analyses testing for selection on the 479-bp cyt b. [file 12862_2023_2155_MOESM1_ESM.docx]

Figure S1. The frequency of the dominating amino acid at each amino acid site of 474-bp *cyt* b (the first amino acid was trimmed, see methods). The plot is based on sequences of all 1,089 avian *Plasmodium* lineages. HPSs are highlighted in pink.


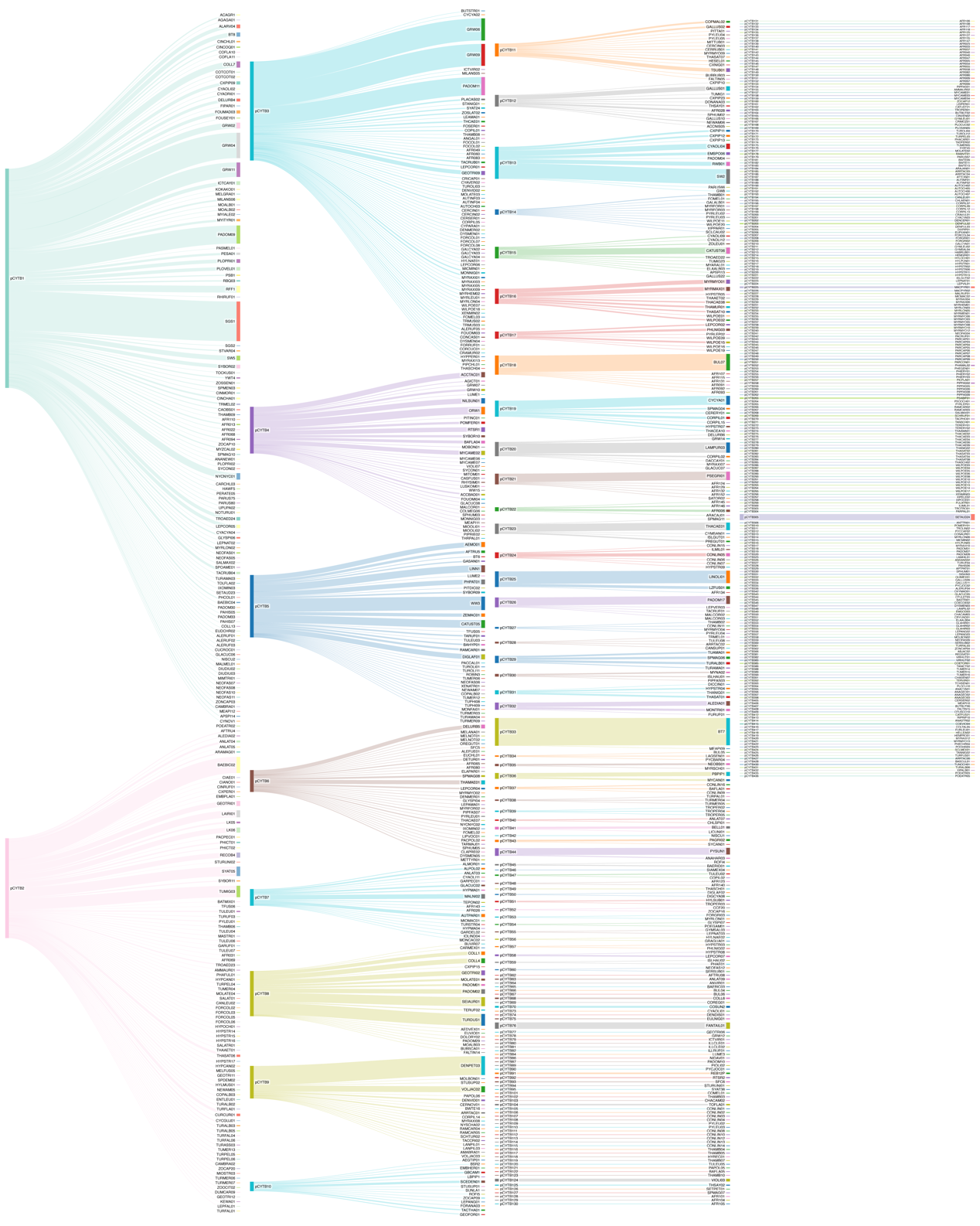


Figure S2. The correspondence between *cyt* b (right side) and their protein haplotypes (left side) of avian malaria parasites. The thickness of the line indicates the size of host range.


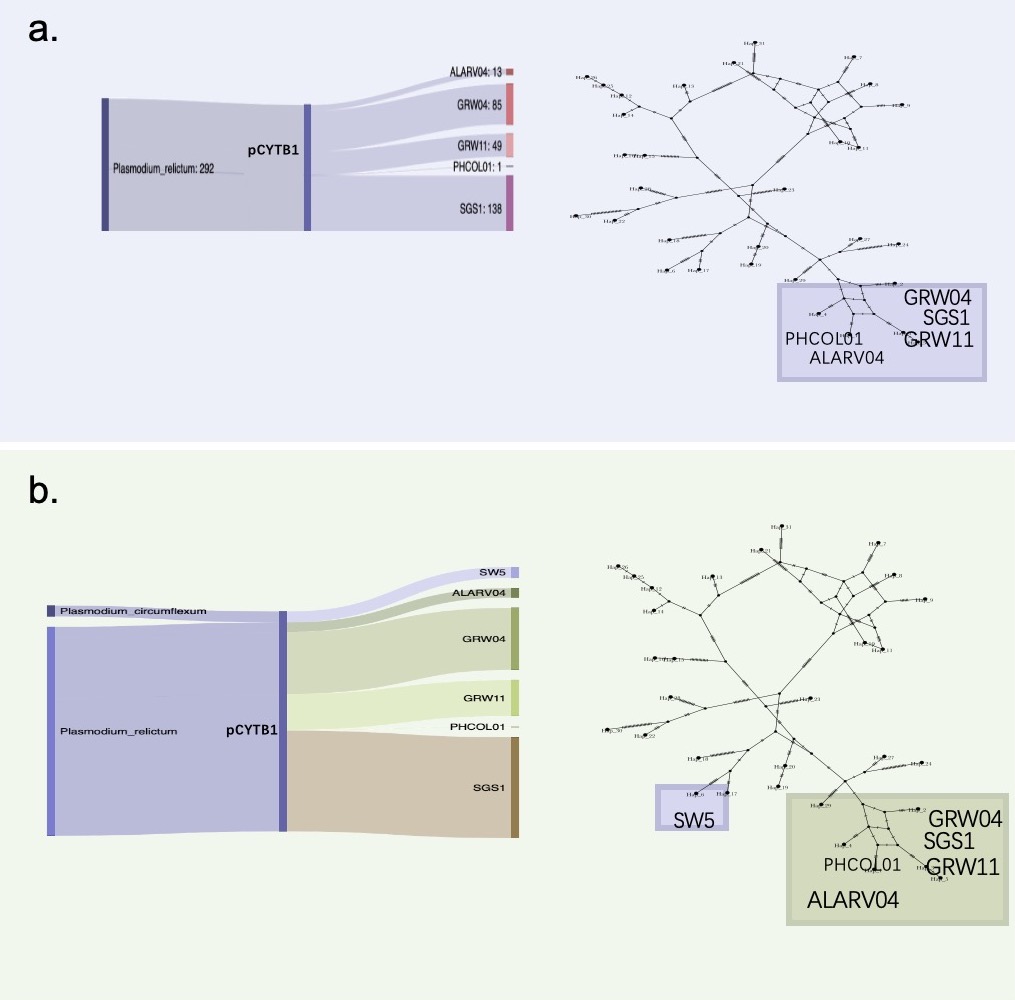


Figure S3 A sub-clade of the avian malaria lineages that share the same protein haplotype (pCYTB1) by phylogenetically related lineages (ALARV04, GRW04, GRW11, PHCOL01, and SGS1) and a distant lineage (SW5).

Table S1 Results of PAML analyses testing for selection on the 479-bp *cyt* b

| **Model** | **Parameter estimates** | **Log likelihood** | **PSSs** | **Model comparison** | ***p(ΔLTR)*** |
| --- | --- | --- | --- | --- | --- |
| M0 | ω0 = 0.02269 | -37130.390 | - | M0 vs M3 | < 0.05 |
| M1a | ω0 = 0.0084 p0 = 0.9432 ω1 = 1.000 p1 = 0.0568 | -35588.611 | - |  |  |
| M2a | ω0 = 0.0085 p0 = 0.9432 ω1 = 1.0000 p1 = 0.0208 ω2 = 1.000 p2 = 0.0356 | -35578.480 | - | M1a vs M2a | < 0.05 |
| M7 | p= 0.17852 q= 1.92845 | -34377.682 | - | M7 vs M8 | < 0.05 |
| M8 | p0=0.99999 p=0.17176 q= 1.59435 (p1 = 0.00001) ω = 2.68953 | -34364.550 | - |  |  |
